# Methodology for Creating a Digital Bathymetric Model Using Neural Networks for Combined Hydroacoustic and Photogrammetric Data in Shallow Water Areas

**DOI:** 10.3390/s24010175

**Published:** 2023-12-28

**Authors:** Małgorzata Łącka, Jacek Łubczonek

**Affiliations:** Maritime University of Szczecin, Waly Chrobrego 1–2, 70-500 Szczecin, Poland; j.lubczonek@pm.szczecin.pl

**Keywords:** digital bathymetric model, big data processing, MLP neural network, data reduction, USV, UAV, data fusion, regression, shallow water area

## Abstract

This study uses a neural network to propose a methodology for creating digital bathymetric models for shallow water areas that are partially covered by a mix of hydroacoustic and photogrammetric data. A key challenge of this approach is the preparation of the training dataset from such data. Focusing on cases in which the training dataset covers only part of the measured depths, the approach employs generalized linear regression for data optimization followed by multilayer perceptron neural networks for bathymetric model creation. The research assessed the impact of data reduction, outlier elimination, and regression surface-based filtering on neural network learning. The average values of the root mean square (RMS) error were successively obtained for the studied nearshore, middle, and deep water areas, which were 0.12 m, 0.03 m, and 0.06 m, respectively; moreover, the values of the mean absolute error (MAE) were 0.11 m, 0.02 m, and 0.04 m, respectively. Following detailed quantitative and qualitative error analyses, the results indicate variable accuracy across different study areas. Nonetheless, the methodology demonstrated effectiveness in depth calculations for water bodies, although it faces challenges with respect to accuracy, especially in preserving nearshore values in shallow areas.

## 1. Introduction

Knowledge of the bathymetric topography in a coastal zone is important in many sectors of the maritime and inland water economy, such as in mapping marine coastal zones, sedimentary processes, near-offshore activities, and environmental protection; therefore, precise mapping of the coastal zone and shallow water areas continues to arouse great interest among researchers, and numerous articles on this subject continue to emerge.

Both hydroacoustic and optical techniques are used for bathymetric mapping. The hydroacoustic method, using multibeam echosounders, is particularly efficient for water bodies that are deeper than double the Secchi depth (SD), and the productivity of this technology increases with increasing water depth because the beam width expands with depth [1]. Although the most reliable information usually comes from hydroacoustic measurements [2], these methods are expensive and do not allow for the mapping of ultra-shallow waters.

Photogrammetry, using structure-from-motion (SfM) and multi-view stereo (MVS), is primarily used due to its low cost and speed in data acquisition. The SfM–MVS method, which uses digital images to acquire bathymetry, can be classified as a passive remote sensing method. There are two approaches in the literature that allow for the acquisition of accurate depth information; the first is based on correction for refraction at the water surface, as presented in publications [3,4,5], and the second assumes the use of an underwater photogrammetric framework [6,7]. Some limitations of this method in mapping the sea bottom include dynamic movement of the water surface and disruptions of the water surface (e.g., white water, ripples, or sun glint), false matching and noisy data [8], and water turbidity, which can occlude the bottom surface and prevent the automatic feature extraction and correspondence steps necessary in SfM for 3D reconstruction. Smooth surfaces with limited texture are difficult for pixel-by-pixel image matching and can result in a less-dense point cloud [8], and water refraction poses significant challenges for depth determination [9]. This method, which can be a source of bathymetric data acquisition, has a known margin of error up to a depth of 1 m [10]. An interesting approach in low-altitude photogrammetry is the use of multispectral sensors [11], which, along with the application of an appropriate algorithm [12,13], allow for the acquisition of bathymetry in shallow water areas with an accuracy of up to 20 cm. Hyperspectral sensors [14] mounted on UAV platforms are also used, and the average errors are less than 13 cm when the water depth ranges from 0.09 m to 1.01 m.

Digital photogrammetry methods are also used to acquire bathymetric data from high-resolution satellite images. Such an approach was presented in [15], in which images from the WorldView-2 satellite were used. The authors of [16] used data from the Landsat 8 satellite to determine the bathymetry of shallow water areas, where depths of up to 7 m were obtained based on the combination of blue–green–red channels. Bathymetry can also be acquired based on active satellite methods; an example of the use of such radar data is shown in [17], which are characterized by moderate resolution and coverage, as well as low reliability in coastal areas.

Another technique that enables data acquisition for mapping shallow waters is LiDAR technology, which is considered an active remote sensing method; it allows for the simultaneous acquisition of data for the morphology of the bottom of water reservoirs while surrounding coastal zone surveillance is carried out from the air [18]. One of the advantages of green LiDAR over hydroacoustic methods is the ability to acquire data in areas that are non-navigable for boats. Much of the attention from research teams is focused on creating inexpensive and effective systems. Such an approach is presented in [19]. The airborne laser bathymetry (ALB) method can also be used for auto-classification and mapping of the seafloor based on machine learning classifiers [20]. The use of LiDAR for acquiring bathymetry is also presented in [21,22,23,24].

Many current studies are related to the fusion of two or more datasets to achieve the best and most uniform representation of the bottoms of water bodies. The authors of the publications combined datasets of the same type, such as in [25], and the method features the integration of separately acquired topographic LIDAR and bathymetric LIDAR data in Port Phillip Bay. Data fusion is most often performed on datasets obtained from different methods, like combining bathymetric datasets and SfM photogrammetry, as presented in [26,27], or bathymetric and satellite data [28]. Also, in the case of satellite techniques, different datasets have been used, as presented in [29], in which a multistep approach leveraging a spectral ratio method of deriving bathymetry from Landsat 8 imagery combined with data from NASA’s airborne ICESat2 ATLAS simulator was used; or in [30], in which the Sentinel-2 and Landsat 8 datasets were combined. Interesting research concerning data fusion is also presented in [31], in which different types of data were combined, including those without geolocation. Additional examples of fusing different datasets can be found in [32,33,34,35,36,37,38,39,40,41].

One of the interesting approaches to data acquisition is the use of neural networks in bathymetric studies. Neural networks can be used to improve the resolution of bathymetric data, and such an approach was proposed in [42]; neural networks were used on images to obtain true depths that are free of the influence of refraction [9,43]; alternately, they were used to create new models that enabled the combination of different datasets [28]. In [44], a neural network was presented that allowed for the acquisition of bathymetric data from optical satellite data, and in [45], networks based on a limited amount of ‘truth’ depths and multispectral data from the Sentinel-2 satellite were presented, allowing for the development of bathymetric maps. Another example of the use of deep neural networks and satellite data can be found in [46].

The application of neural networks requires the preparation of a training sequence on the basis of which the network can be trained, and in the final phase, a model can be created. The authors of this study investigated the possibility of using partial optimization of the training set to reconstruct the bathymetric surface created from hydroacoustic and photogrammetric data; this constitutes a new contribution to the field of research related to the creation of hybrid surfaces in shallow water areas. Partial optimization is the result of the combined overlap of hydrographic and photogrammetric measurements. Another aspect of the research is the method of fusing data from unmanned surface vehicles (USVs) and unmanned aerial vehicles (UAVs). From the conducted research and proposed data processing methodology, a new approach to point cloud filtration using generalized linear regression [47] was proposed after conducting a series of different experiments, with the elimination of outliers or a reduction in the measurement points in the dataset.

The main aim of the research was to develop a methodology for creating digital bathymetric models (DBMs) that use multilayer perceptron (MLP) neural networks. The research concerns the specific case in which it is assumed that the training dataset covers only a part of the depth data. The order of research is related to the optimization of the data used to create the datasets for learning neural networks. This part of the research brings to realization the following sub-goals:

- The impact of eliminating outlier data on the learning of the neural network;
- The impact of dataset reduction on the learning of the network;
- The impact of regression surface-based filtering on the learning of the neural network;
- Using neural networks to create bathymetric surfaces from fusing hydroacoustic and bathymetric data.

Due to the complexity of the research, one architecture of neural networks was focused on to allow for in-depth research analysis. The obtained results will certainly be a useful reference for further research with other types of neural networks. 

The remainder of the article includes the following sections: In Section 2, the study area and the datasets used are presented. In Section 3. The methodology and the entire research process are presented. In Section 4, the obtained results are presented, and the quantitative and qualitative analyses are described. In Section 5, the discussion compares the obtained neural digital bathymetric model (NDBM) results to those of other methods, and the final conclusions are stated.

## 2. Study Area and Dataset Preprocessing

**Figure 1:**
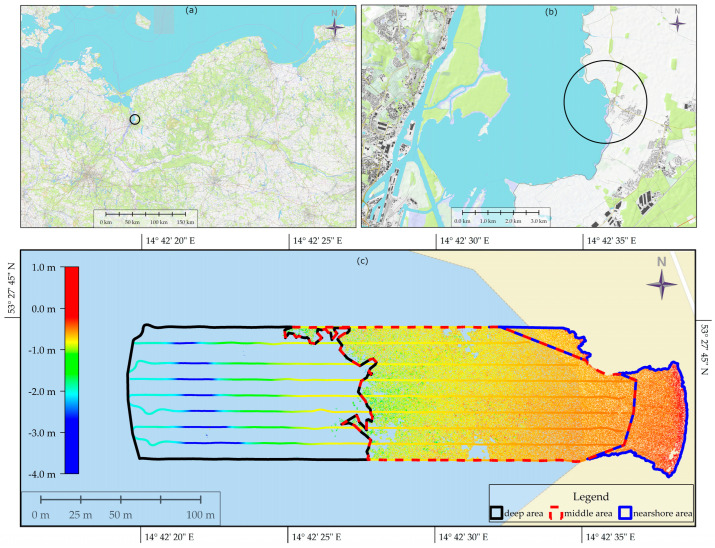
The black circle (**a**,**b**) marks the study area that includes a section of Lake Dabie located in Czarna Laka. The study area (**c**) and the studied hydroacoustic and photogrammetric datasets and depths are presented on a hypsometric scale [7]. The study area is divided into deep, middle, and nearshore areas, and the extents of these areas are marked by black, red, and blue lines.

The research area was located within the village of Czarna Laka (Poland), situated in the West Pomeranian Voivodeship in Goleniow County. The research area is presented in Figure 1; its surface area amounts to 2.71 hectares. The conditions for data acquisition can be considered favourable; the water turbidity, colour, riparian vegetation, and weather conditions had an insignificant impact on the measurements performed [7].

### 2.1. UAV Dataset—Photogrammetric Point Cloud

The data were acquired using the unmanned aerial system DJI Phantom 4 Pro. The flight was carried out at an altitude of 120 m using a UV filter, during which 439 photos were taken in 12 rows. A signalized photogrammetric network consisting of ten ground control points (GCPs), six terrestrial and four underwater, was used to align the images. The GCPs were measured using a Sokkia GRX-1 receiver operating in real-time kinematic positioning (RTK) mode. The processing was performed with PIX4D Mapper software (v. 4.4.12). Based on the photos, a dense point cloud was generated. A classification of the point cloud was also carried out, separating points representing the ground according to the algorithm presented in [48]. To georeference the photos, a terrestrial and underwater framework was used without corrections for refraction. The values of the obtained RMS errors were X = 0.005 m, Y = 0.013 m, and Z = 0.057 m. For further processing, only part of the point cloud representing ground points was used. The photogrammetric input set consisted of 2,491,923 points, the maximum value in the set was 0.86 m, the minimum value in the set was −3.6 m, the mean value was −0.7 m, and the standard deviation was 0.19 m. The obtained photogrammetric point cloud is a high-roughness representation of the bottom of the studied water area [7].

### 2.2. USV Dataset—Single-Beam Echosounder Data

The data were acquired using a remotely controlled floating platform, which is most often used for measurements on extremely shallow waters. The measuring system consisted of a single-beam echosounder, Echologger EU400, equipped with an inertial measurement unit (IMU) for correcting platform movement. The echosounder is characterized by a high acoustic frequency and the small size of the acoustic beam. In the process of data acquisition, 6816 points representing the bed were acquired in nine measurement profiles. The minimum depth in the set was 0.48 m, and the maximum depth was 3.95 m. Subsequent survey profiles, shown in Figure 1, were about 10 m apart, and their lengths ranged from 250 to 300 m. The acquired dataset was processed in the SBMAX64 module of Hypack software (version 2021), and analysis of the echograms was carried out manually. The hydroacoustic data were vertically referenced to a water-level gauge located at the Most Długi in Szczecin. Based on the set of hydroacoustic data, the bathymetric reference surface (BRS) was created using the triangulated irregular network (TIN) method. Detailed information regarding data acquisition is presented in article [7].

## 3. Methodology

**Figure 2:**
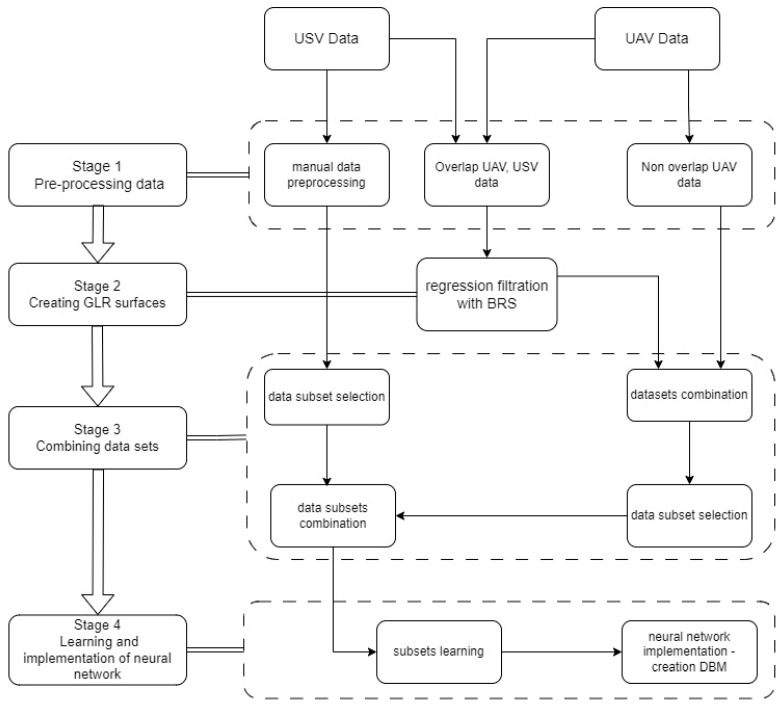
Diagram of merging photogrammetric and hydroacoustic data to create a digital bathymetric model.

The diagram presented in Figure 2 shows the process of combining hydroacoustic and photogrammetric data in order to build a digital bathymetric model. Later in the article, the following acronyms are used for the study area, which is divided into three parts: nearshore area—SH (only photogrammetric point cloud data), middle area—OV (single-beam echosounder and photogrammetric point cloud data), and deep area—DE (single-beam echosounder data). The research methodology consists of 4 stages. The first stage is the pre-processing of the acquired source data. The second stage is the creation of regression surfaces. Stage 3 includes the merging of datasets. The final stage, stage 4, presents the process of training the MLP network and the obtained results. The diagram presented in Figure 2 illustrates the main stages of processing individual datasets in order to obtain a uniform digital bathymetric model throughout the entire area being developed.

### 3.1. Data Pre-Processing (Stage 1)

#### 3.1.1. Pre-Processing Non-Overlap Photogrammetric Point Cloud Data

The nearshore dataset consists of a point cloud acquired after processing UAV images, located outside the USV data coverage area. A component of the experiment in this stage was point reduction, which enabled the subsequent investigation of its effect on surface reconstruction. This set was reduced using the reduce point density tool [49] with four reduction ratios of the following sizes: 10 cm, 25 cm, 50 cm, and 100 cm. The result was a lower-density dataset consisting of evenly distributed points in the UAV data domain. The reduction was based on the elevation attribute. Table 1 presents the statistics of the original coastal dataset and the reduced datasets. As the ratio of reduction increases, the number of points decreases significantly. In quantitative terms, a significant decrease in the number of points can be observed with a larger reduction ratio, which are for ratios of 10 cm—58%; 25 cm—90%; 50 cm—97%; and 100 cm—99%. It should be noted that despite the significant quantitative reduction, the measurement set still consists of a large number of points, from 3221 to 183,204.

#### 3.1.2. Pre-Processing Overlap Single-Beam Echosounder and Photogrammetric Point Cloud Data

This section presents a methodology for reducing the photogrammetric dataset based on a bathymetric reference surface, the creation of which is described in detail in publication [7]. This area was covered by both hydroacoustic and photogrammetric data. In the first processing step, the D_BRS_ deviation between the BRS surface and each point belonging to the photogrammetric collection was calculated. If P_UAV_ means the set of points in the UAV point cloud, where *n* is the number of points in the following:

P_UAV_ = {p_1_, p_2_,..., p_n_} (1)

then for each point *p_i_*, the deviation of D_BRS_(p_i_) can be expressed by the following formula:

D_BRS_(p_i_) = |Z_BRS_(p_i_) − Z_UAV_(p_i_)| (2)

where Z_BRS_ is the depth represented by the BRS surface and Z_UAV_ is the depth represented by the photogrammetric measurement points. The set of D_BRS_ deviation values for all photogrammetric measurement points relative to the BRS surface can be written as follows:

D_BRS_ = {D_BRS_(p_1_), D_BRS_(p_2_),..., D_BRS_(p_n_)} (3)

The set of overlapping hydroacoustic and photogrammetric data in the subsequent processing steps was filtered using D_BRS_ deviation via statistical, data reduction, and linear regression methods to select the optimal processing parameters. The full processing of the original photogrammetric set from the overlap area was performed according to the following steps, 1–4, as shown below:

1. Reducing outlier observations;
2. Reducing statistical sets;
3. Filtering the reduced sets;
4. Creating the DBM based on the MLP neural network.

These stages are described below.

#### 3.1.3. Reduction in Outliers

The statistical sets were separated based on the D_BRS_ parameter, for which the mean *(m)* and standard deviation (*σ*) were calculated. On the basis of these parameters, four new sets were created, where the threshold value for reduction was the parameter *t*. The value of this parameter is the sum of the value of *m* and the corresponding multiplicity of the standard deviation:

t_1_ = m + 0.5σ (4)

t_2_ = m + 1σ (5)

t_3_ = m + 2σ (6)

t_4_ = m + 3σ (7)

The resulting sets, S_1_, S_2_, S_3_, and S_4,_ can be written as follows:

S_1_ = {p_i_ ∈ P_UAV_ | D_BRS_(p_i_) < t_1_} (8)

S_2_ = {p_i_ ∈ P_UAV_ | D_BRS_(p_i_) < t_2_} (9)

S_3_ = {p_i_ ∈ P_UAV_ | D_BRS_(p_i_) < t_3_} (10)

S_4_ = {p_i_ ∈ P_UAV_ | D_BRS_(p_i_) < t_4_} (11)

The statistics of the sets are presented in Table 2. The statistical sets were expected to remove outliers. In this case, the sizes of the statistical sets did not change significantly from the original dataset and represent 82%, 89%, 96%, and 98% of the input sets, respectively.

#### 3.1.4. Analysis of Options for the Reduction in Statistical Sets

**Figure 3:**
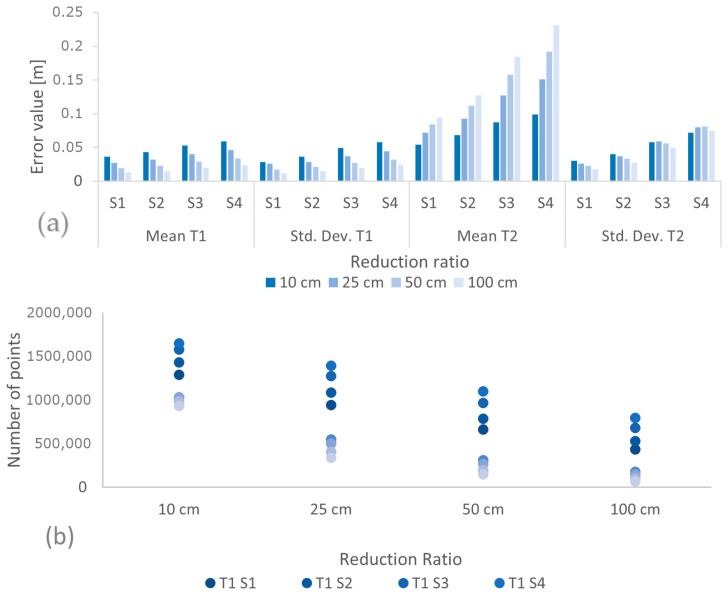
Summary of photogrammetric point cloud reduction results: (**a**) mean values and standard deviations, (**b**) number of points in datasets.

**Figure A1:**
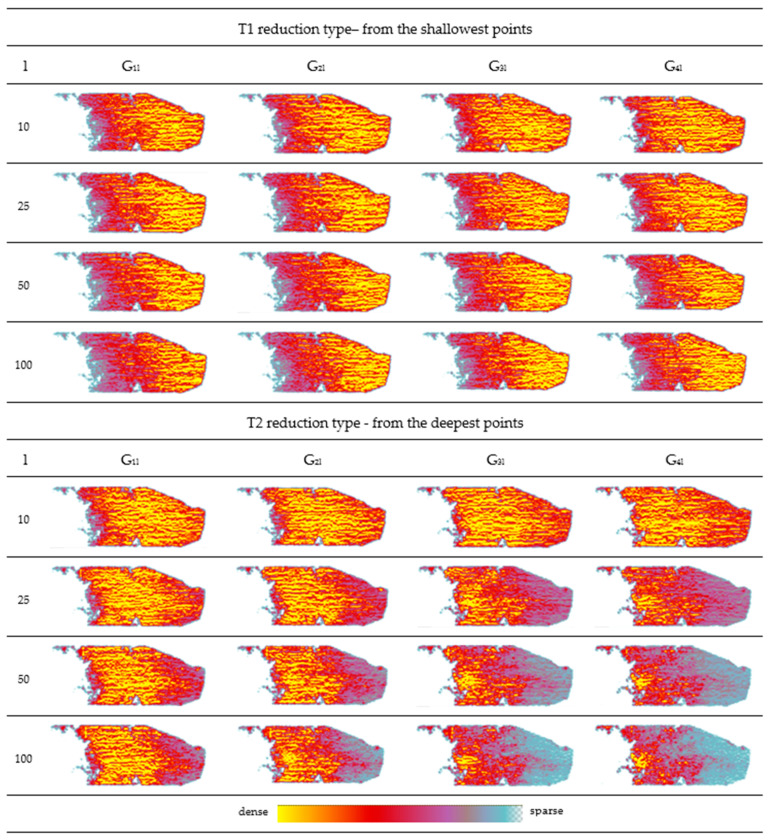
Point clouds shown as density maps obtained in stage 1 (from reducing the statistical set).

The next stage of data processing consisted of reducing the statistical sets using the reduce point density tool [9] available in ArcGIS Pro software (v. 2.9.0). The parameters necessary for the tool to work are the size of the reduction ratio and the direction, from which the points are reduced. The following reduction ratios (l) were used in the study: 10 cm, 25 cm, 50 cm, and 100 cm, as well as reductions from the shallowest (T1) and deepest (T2) points. The result of this tool on previously prepared statistical datasets is 16 new datasets for each type of reduction, for a total of 32 new datasets. The results obtained representing the datasets in the form of point density maps are presented as Figure A1 (Appendix A). Based on the qualitative evaluation for the T1 reduction, the 16 datasets obtained presented in the form of a density map do not show large differences between the datasets obtained; the most noticeable changes in the density of points are visible on the right where the deepest points appear. Based on the qualitative analysis for the T2 reduction, there is a rapid decrease in point density in the nearshore area with an increasing reduction ratio. For the 32 datasets, a quantitative analysis was performed on the bases of the mean value ${\bar{D}}_{BRS}$, the standard deviation, and the number of points. The results are summarized in graphs (Figure 3).

On the basis of the graphs shown in Figure 3a,b, the following can be concluded:

1. The mean values of the D_BRS_ assume higher values for the T2 reduction type;
2. The mean values of the D_BRS_ for T1 reduction take a maximum value of 0.06 m for set S_4_ with a reduction radius of 10 cm;
3. For sets with a T1 reduction type, the values of the mean and standard deviation decrease in each statistical set; for T2 reductions, as the reduction ratio increases, the values of the mean significantly increase in the statistical sets, and the value of the standard deviation decreases in the statistical sets;
4. T2 reduction type provides smaller datasets.

Based on the analysis of the results obtained, the sets from the T1 reduction were used for further studies. The increasing mean error for the T2 reduction type, up to a maximum value of 0.23 m in the case of a 100 cm reduction, is almost four times higher than the maximum error (0.06 m) for the T1 reduction type; on this basis, it was decided not to continue processing on the T2 reduction type set.

### 3.2. Creating GLR Surfaces (Stage 2)

**Figure 4:**
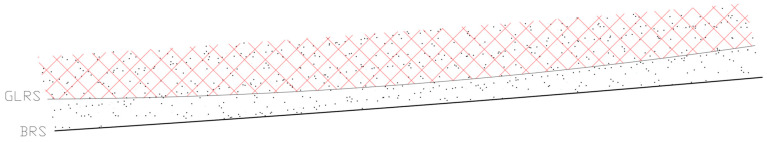
Point cloud filtering process based on regression surfaces. Points from the photogrammetric point cloud dataset that are above the regression surface (GLR) are removed (hatched area with red lines).

The next data processing step was to create filter surfaces based on the generalized linear regression tool and perform further data reduction. The tool is available in ArcGIS Pro software (v. 2.9.0) and performs generalized linear regression [50]. A regression surface was created for each dataset. The purpose of the regression surface was to filter out the points from the photogrammetric point cloud (Figure 4). If we label the depths in the same coordinates (x_i_,y_i_) of the P_UAV_ points on the GLRS regression surface Z_reg_ as follows:

Z_reg_ = {p_1_, p_2_,..., p_n_}, where *n* is the number of points. (12)

and the set of point reduction parameters (*l*) expressed in centimeters as L = {10, 25, 50, 100}, then the filtered sets can be described as follows:

G_kl_ = {p_i_ ∈ S_k_ | D_BRS_(p_i_) − Z_reg_(p_i_) ≤ 0}, where k = 1,2,3,4 and l ∈ L. (13)

**Figure 5:**
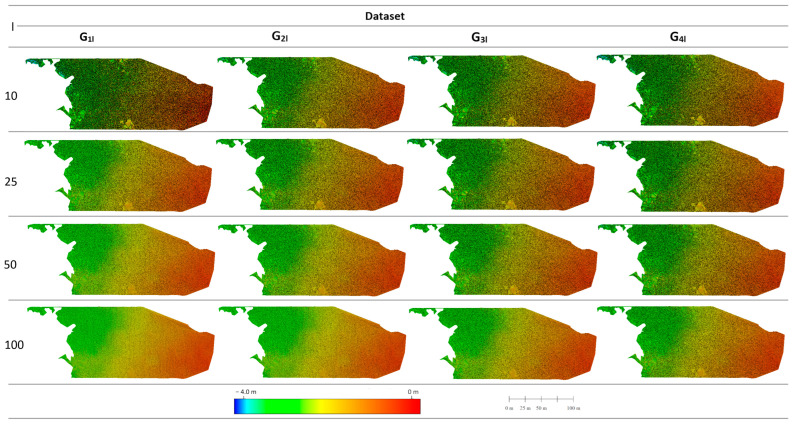
Overview of the surfaces representing the middle area created from the filtered point cloud.

From the 16 sets of filtered photogrammetric point clouds, 16 surfaces were created to represent the middle part (Figure 1) of the study area (Figure 5). Performing a visual assessment of these surfaces on the basis of surface roughness allows the filtering effect to be assessed. The smoothest surface obtained is the G_1l_ collection for l = 100, and the roughest surfaces for the parameter l = 10 cm. For the parameter l = 50, the surfaces G_1l_ and G_2l_ are characterized by significantly less roughness, which may indicate a stronger filtering influence for these surfaces. A weaker filtering effect will be observed for l = 10 and l = 25 as well as for the G_3l_ and G_4l_ surfaces.

### 3.3. Creation of Training Datasets (Stage 3)

**Figure 6:**
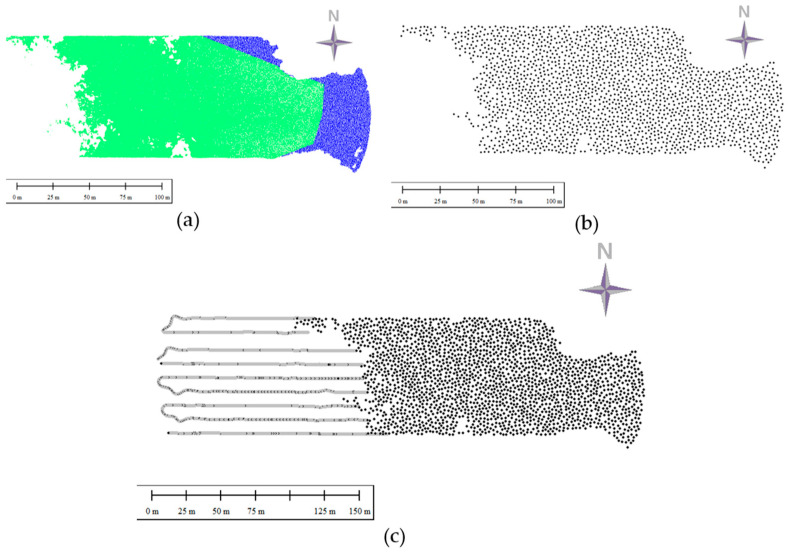
Merging process of datasets: (**a**) merging of the middle (green) and nearshore (blue) datasets; (**b**) reduction in points from the middle and nearshore parts using the create random points tool; (**c**) merging of the reduced dataset with the hydroacoustic data.

The process of creating the training datasets involved several steps. First, the middle and nearshore sets were combined (Figure 6a). The merging was performed on the basis of the compatibility of sets with the same reduction ratio. Then, from each combined set, a collection of 2000 points was sampled (create random points tool, ArcGIS Pro, v.2.9.0) (Figure 6b). This reduction in the number of points in the set was intended to create an optimal training dataset for neural network learning. An important condition for creating the training dataset was a uniform distribution of the points, so the condition of minimum distance between neighboring points was applied. In order to allow the full reference surface to be generated, points from bathymetric surveys were added to the sampled sets (Figure 6c). The training sets prepared in this way were used in Statistica (v. 13.3) to train the neural networks. The whole process of merging points is illustrated in Figure 6.

### 3.4. Creation of Neural Digital Bathymetric Models (Stage 4)

The same settings for neural network generation were used for each set. Regression multilayer perceptron neural networks were used. The network architecture consisted of an input layer, an output layer, and one hidden layer. The logistic function was used as the function for the input neurons and the linear function as the output. For each set, 1000 networks were created, of which the best 50 were saved for analysis. The training set was divided into the following subsets: 75% training set; 25% test and validation set. Table 3 summarizes the results obtained for the training sets. As quality parameter (Table 3), the Pearson correlation coefficient was marked in Statistica (v. 13.3) with a division into training and test and validation sets; this parameter informs about the predictive strength of the created network [51]. The network name includes the number of hidden neurons used to create the network; the maximum number of hidden neurons was set to a value of 30. The quasi-Newton BFGS algorithm [52,53,54,55] was used to teach the MLP network. This algorithm takes advantage of the fact that a direction to the minimum can be found on the quadratic error function using a second-order partial derivative matrix [56]. The sum of squares (SOS) was used as the error of the function, which is a measure of error equal to the sum of the squares of the differences between the predicted (by the model) and actual (observed) values [56].

The next step was to create a bathymetric surface using the created neural models. The models were generated in a GRID structure with a resolution of 1 m, resulting in the calculation of 27,208 depth values in the raster cells. The neural digital bathymetric models created in this way were evaluated further.

## 4. Results

### 4.1. Test Dataset

**Figure 7:**
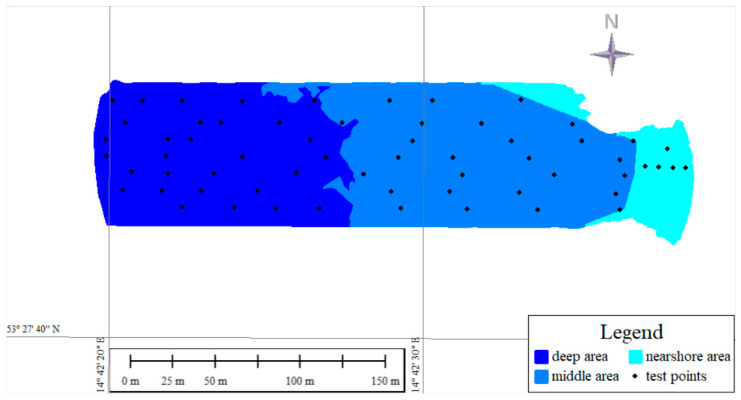
Distribution of test points.

The test dataset consisted of 61 points (n) that were measured using the hydroacoustic (56 points) or GNSS-RTK technique (5 points) and did not participate in previous processing; these points were a separate independent dataset. The test dataset, like the data processing area, was divided into three parts: nearshore, middle, and deep. The nearshore part contained five test points; the middle part, twenty-eight points; and the deep part, also twenty-eight points. The distribution of the test points and the processing area is shown in Figure 7. The statistics for the set were as follows: maximum value of −0.47 m, minimum value of −3.92 m, mean value of −1.29 m, and a standard deviation of 0.87.

### 4.2. Qualitative Assessment

**Figure 8:**
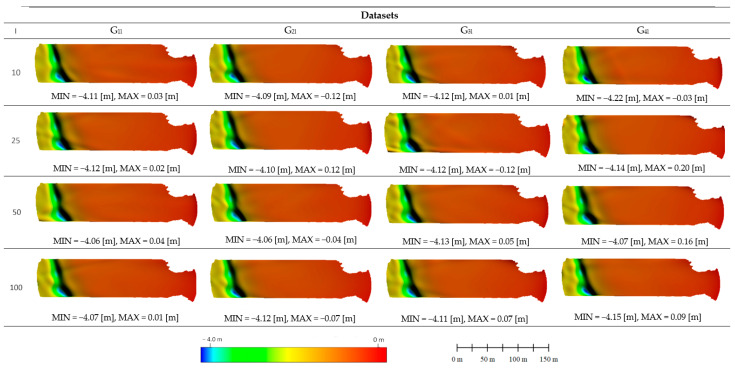
Summary of NDBMs obtained for the studied cases: statistical and reduced. MIN and MAX represent the minimum and maximum depth values on the NDBMs, respectively.

Figure 8 summarizes the surface representations of the neural digital bathymetric models (NDBMs) generated by the MLP neural networks. All of the surfaces created, regardless of the statistical set or level of reduction used, were quite similar, especially in the nearshore and middle parts, which are areas that are relatively flat and characterized by a slight gradual decrease in depth. The greatest changes occur in the deepest part within the local depression, where it is easiest to see the differences in the created NDBMs. A characteristic feature of the created NDBMs is the lack of surface roughness; this is a result of the processing methodology and the pixel size used (1 m). The consequence of this approach is the inability to recognize bottom changes smaller than the size of the pixel. Figure 8 also summarizes the minimum and maximum depths for the created surfaces. The minimum values obtained on all models are in a similar range and extend from −4.22 m to −4.06 m. The maximum values on the models are characterized by a much larger distribution; the highest maximum value was obtained for the set G_4l_, l = 25 with the value of 0.20 m, and the lowest maximum value was obtained for the set G_2l,_ l = 10 with the value of −0.12 m.

### 4.3. Datasets for Results Analysis

The final 16 NDBMs (Figure 8) were obtained through data cleaning, reduction, and filtering. In addition, five new reference neural digital bathymetric models (rNDBMs) were created for the quantitative analysis process. These sets were created without steps 1 (outlier removal), 2 (data reduction), and 3 (filtering).

The original dataset was used to create the first reference dataset, without any processing (UAV PC). To generate the subsequent four rNDBM reference sets, a combination of nearshore and middle sets was utilized. These sets were exclusively based on data reduction using the depth parameter (depth). The same reduction ratio values as those applied in the previous data filtering process were used, with data sizes of 10 cm, 25 cm, 50 cm, and 100 cm. The rNDBM sets prepared in this way were analyzed quantitatively on the test dataset in order to compare the differences between the models.

### 4.4. Results

For each test point, the discrepancy between the surveyed (Z_i_) and mapped depths (z_i_) was determined for each of the 16 DBMs created. Subsequently, the mean absolute error (MAE) was calculated as per Equation (14). Additionally, the root mean square error (RMSE) was computed, representing the error in terrain plasticity, which serves as a measure of fit, as outlined in Equation (15). Finally, the maximum absolute error (MaxAE) was determined in accordance with Equation (16).

(14) $$\mathrm{MAE}=\frac{\sum_{i=1}^{n}\left|Z_{i}-\left.z_{i}\right|\right.}{n}$$

(15) $$\mathrm{RMSE}=\sqrt{\frac{\sum_{i=1}^{n}(Z_{i}-z_{i}{)}^{2}}{n}}$$

(16) $$\mathrm{MaxAE}=\underset{i\in \left\{1,2,\ldots n\right\}}{\max}\left|Z(i)-z(i)\right|$$

**Figure 9:**
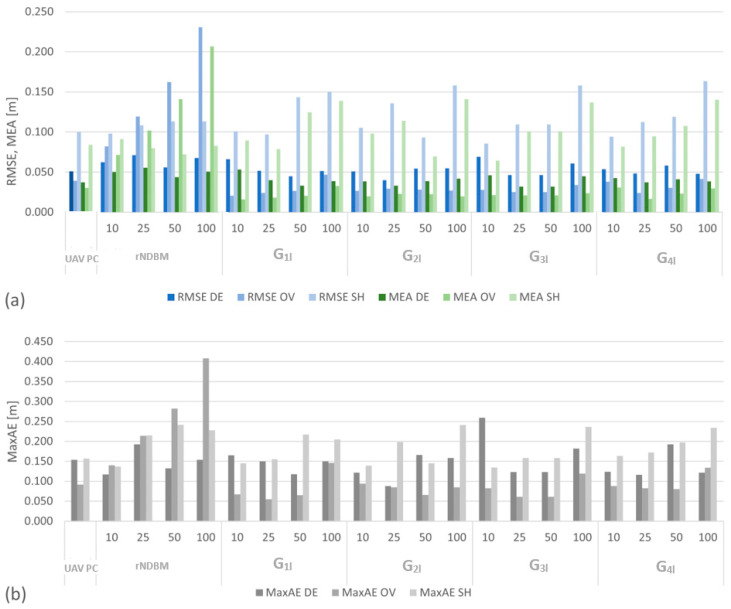
Summary of (**a**) RMSE and MEA; (**b**) MaxAE—for the cases studied: reduced and statistical, divided into nearshore (SH), middle (OV), and deep (DE) areas.

The graph shown in Figure 9 summarizes the MAE, RMSE, and MaxAE values obtained for the five reference surfaces (first two sections of the graph: UAV PC and rNDBM) and for the 16 filtered NDBMs. The MAE and RMSE are displayed using a grouped column graph and a green and blue color gradient, with the color intensity varying according to the section. The MaxAE are presented as dot plots.

Analyzing the MAE, it can be observed that the largest values of this error occur for the middle part (MAE OV) on the rNDBMs, while in the remaining 16 NDBMs for this part, the values of the MAE error are considerably lower than the values of this error for the nearshore part (MAE SH) and the deep part (MAE DE). The MAE assumes the largest values for the nearshore part (SH) in the filtered sets, regardless of the reduction ratio and the statistical set. For the deep part (DE) and filtered sets, the MAE always has values larger than those on the middle part (OV); a similar trend of this error can be observed for the errors obtained for the original set (UAV PC).

In the middle part (OV) for the 16 NDMBs, the value of the RMS error is always smaller than its value in the nearshore part (SH) and in the deep part (DE). In the case of the four rNDBMs, the value of the RMSE for the middle part (OV) visibly increases with an increasing reduction ratio, from a value of 0.08 m for a reduction ratio of 10 cm to a value of 0.23 m for a reduction ratio of 100 cm. In the middle part (OV) for the 16 filtered datasets, the value of the RMS error is always smaller than its value in the nearshore part (SH) and in the deep part (DE). The value of the RMS error for the original set is 0.04 m; for the filtered sets, larger error values occur on two sets for the parameter l =100 (G_1l_ and G_2l_).

Analysis of the MaxAE for the middle part (OV) shows for the filtered sets that the MaxAEs occurring on the central part are lower for each set than for the MaxAEs on the nearshore (SH) and deep (single-beam echosounder) parts. This indicates the positive effects of the filtering performed with the use of BRS surfaces.

On the rNDBM set for the middle area (OV), an increase in the values of the MAE, RMSE, and MAX errors is visible as the reduction ratio increases. For the nearshore (SH) and deep (DE) areas, such systematic error increases are not noticeable.

**Figure 10:**
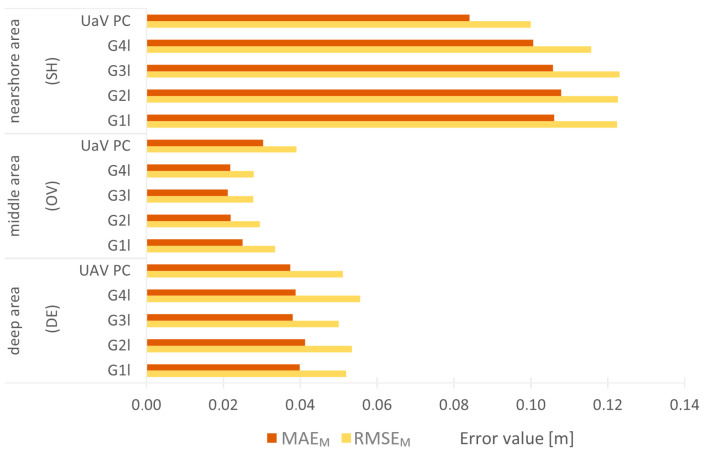
Summary of MAE_M_ and RMSE_M_ for the nearshore, middle, and deep areas.

The RMSE and MEA did not show large variations in G_1l_, G_2l_, G_3l_, and G_41._ For further quantitative analyses, these errors were averaged according to Formulas (17) and (18), where *m* denotes the number of reduction levels tested; the results are summarized in Figure 10.

(17) $${MEA}_{M}=\frac{MEA}{m},$$

(18) $${RMSE}_{M}=\frac{RMSE}{m},$$

The chart is divided into three sections, with MAE_M_ and RMS_M_ errors summarized for each processing area. The RMSE_M_ has a high similarity within each section showing the deep, middle, and coastal areas. The smallest errors occur in the middle section and do not exceed 0.03 m. The largest error is on the nearshore part, where the value is 0.12 m. On the deep part, the largest value of the RMS_M_ error is 0.06 m.

The MAE_M_ errors also assume the smallest value for the middle part of 0.02 m. The largest value for the nearshore part is 0.11 m, with a value of 0.04 m for the deep part. Comparing the average of the RMSE and MAE for the statistical sets and the original set of the UAV PC, it can be seen that for the middle set, these errors take values larger for the original cloud than for the subsequent filtered datasets. Regardless of the assumed statistical set, it can be observed that the filtered middle part has the smallest error values.

The values of the MAE_M_ from the deep part, relative to those from the middle area, are multiples from 1.6 to 1.9, while the relative values of the RMSE_M_ are from multiples of 1.5 to 2. Comparing the errors between the coastal and middle areas, the MAE_M_ are from multiples of 4.2 to 4.9, while the RMSE_M_ are from multiples of 3.7 to 4.4.

## 5. Discussion

The conducted research aimed to determine whether preparing a training set for neural network learning, based only on a part of the generated surface, would allow for the correct reconstruction of the full range of data acquisition for USV and UAV in shallow and ultra-shallow inland water areas. It was examined whether and how the accuracy of modelling is affected by filtering the point cloud using statistical parameters (mean value and standard deviation) and data reduction. During the research, 16 cases were examined for which the bottom surfaces were calculated, and qualitative and quantitative analyses were performed.

Based on the obtained results and the comparative analysis performed, it can be stated that the optimization performed using the D_BRS_ parameter and GLR allowed NDBMs to be obtained whose accuracy varies depending on the surface part (middle, deep, or nearshore). The RMS error is four times greater for the nearshore part than for the middle part and over twice as large for the deep part. The least accurate results occur in the nearshore part, where the only depth information is obtained from the photogrammetric point cloud. The middle part, which had dual information—hydroacoustic and photogrammetric—was created most accurately, and the optimization of the set is reflected in the small error values in this area (RMSE = 0.03 m, MEA = 0.02 m). The tested multi-variant cases, based on statistical sets and their data reduction, allow us to conclude that increasing the reduction ratio, and thus reducing the dataset, can lead to incorrect data modelling (increasing the MAE). The analysis of the MaxAE in subsequent sets allowed us to observe that the largest values are the MaxAEs for the nearshore part. It can also be stated that the size of the MaxAE for the nearshore part increases with the reduction ratio.

Comparing the error sizes of the MaxAE, RMSE, and MEA as percentages in relation to the original dataset, it can be stated that for the middle part (OV), 88% of the RMSEs and MEAs and 75% of the MaxAEs are smaller for the optimized sets. For the deep part (DE), 38% of the RMSEs, 25% of the MEAs, and 75% of the MaxAEs are smaller than the corresponding errors on the original set. For the nearshore part (SH), there is an improvement of 31% for the RMSE, 44% for the MEA, and 38% for the MaxAE. The analysis of the results allows us to state that in the case of each area, an improvement was achieved that reduced the errors, but it was not uniform in the aspect of the analysed parts of the NDBMs. 

Comparing the presented results with the accuracy obtained using the research methodology presented in [7], using selected interpolation methods for the same dataset, the following error was obtained: RMS = 0.03 m. It can be stated that the error sizes in the proposed research methodology were achieved only for the middle part. In relation to other, similar studies for very shallow and turbid tidal environments, such as in publication [42], the errors obtained were MEA = 0.05 m and RMSE = 0.18 m. For comparison, one can also mention the accuracies obtained using LiDAR technology for mapping shallow water areas [25], where MEA = 0.0035 m and RMSE = 0.04 m were achieved.

An interesting observation was the ability to preserve the real values of depths and the possibility of eliminating outliers, which may appear in nearshore areas. Research in this area was conducted in [57] using surface filters. Smoothing the surface with filters caused a slight artificial shallowing of the water body and the elimination of outliers in the coastal strip. In the case of the neural network, on the other hand, slightly greater depths were obtained. In relation to the greatest depth from the measurement set, in the NDBMs, the depths were always greater by values ranging from 11 to 27 cm. Moreover, the neural network did not eliminate outliers in the area where water and land met. This is certainly an important fact from an application perspective, because for navigational purposes, the calculated depths cannot be greater than the real ones due to the possibility of a ship running aground. In this case, there is a possibility of not meeting certain hydrographic accuracy requirements, e.g., for special areas [58]. In terms of the applicability of the model, this seems to be a significant limitation of the method.

## 6. Conclusions

Based on the study, the following conclusions were drawn:

- The final accuracy of the calculated depths varies for each of the examined areas and depends on the input dataset;
- The filtering performed using the linear regression allowed the removal of outlier observations for the middle area;
- The reduction in the multi-million photogrammetric dataset is a most important step in creating a learning set;
- MLP neural networks allow depth calculation but may not preserve the true boundary values; the usefulness of such obtained models for navigation purposes may be limited, especially in shallow water areas.

The obtained results also confirm the correctness of the hypothesis posed; however, they indicate the increased efficiency of the used neural network models in parts lying outside the BRS surface: for example, by applying networks of a different architecture or type.

An advantage of neural networks containing up to 30 hidden neurons is the ability to process data quickly without the need for powerful workstations, as well as the ease of their implementation into a chosen spatial grid. This makes it possible to efficiently create and implement neural networks. In the conducted studies, 1-meter grids were used for the deployment of the networks. The weakness of the proposed method is its inability to reproduce details of the seabed that are smaller than the spatial resolution of the created rasters and the lack of the preservation of extreme depth values.

The study also demonstrated that reducing a multi-million, densely populated dataset was necessary to obtain an efficient dataset for training the neural network. Considering the implementation of the research process, it is important to note that data reduction is one of the more significant factors in deploying neural networks. Based on the conducted studies, the authors have identified potential areas for further research in creating digital bathymetric models from hybrid data.

## Figures and Tables

**Table 1:** Statistics of reduced dense point cloud sets.

	Input Dataset	Reduction Ratio			
		10 cm	25 cm	50 cm	100 cm
Number of points	438,843	183,204	45,556	12,868	3221
Maximum value (m)	−1.7	−1.7	−1.1	−1.0	−0.7
Mean value (m)	−0.4	−0.4	−0.4	−0.4	−0.3
Standard deviation (m)	0.16	0.16	0.16	0.17	0.17

**Table 2:** Summary of statistical sets.

Dataset	S_1_	S_2_	S_3_	S_4_
Number of points	1,679,496	1,836,494	1,972,763	2,021,762
Max value (m)	0.12	0.16	0.25	0.335
Mean value (m)	0.04	0.05	0.06	0.07
Standard deviation (m)	0.03	0.04	0.05	0.06

**Table 3:** List of the neural networks used to create the digital bathymetric models.

	Reduction Ratio (l) [cm]	Neural Network Name	Quality (Learning)	Quality (Testing)	Quality (Validation)	Learning Algorithm	Error Function
G_1l_	10	MLP 2-30-1	0.997579	0.997687	0.997110	BFGS 1286	SOS
	25	MLP 2-28-1	0.998315	0.998366	0.998245	BFGS 1115	SOS
	50	MLP 2-29-1	0.998106	0.997959	0.997642	BFGS 1516	SOS
	100	MLP 2-28-1	0.998130	0.997679	0.998229	BFGS 1075	SOS
G_2l_	10	MLP 2-30-1	0.997940	0.998245	0.998116	BFGS 844	SOS
	25	MLP 2-28-1	0.998107	0.998055	0.998017	BFGS 1074	SOS
	50	MLP 2-30-1	0.998215	0.998124	0.998015	BFGS 1132	SOS
	100	MLP 2-29-1	0.998046	0.998050	0.998328	BFGS 944	SOS
G_3l_	10	MLP 2-30-1	0.997976	0.998204	0.997950	BFGS 1370	SOS
	25	MLP 2-29-1	0.998249	0.998507	0.997931	BFGS 1227	SOS
	50	MLP 2-28-1	0.998282	0.997973	0.997875	BFGS 1307	SOS
	100	MLP 2-30-1	0.998332	0.998146	0.998344	BFGS 1126	SOS
G_4l_	10	MLP 2-27-1	0.997582	0.997774	0.997705	BFGS 1113	SOS
	25	MLP 2-30-1	0.998351	0.998232	0.998221	BFGS 1399	SOS
	50	MLP 2-28-1	0.998390	0.998508	0.998470	BFGS 1323	SOS
	100	MLP 2-30-1	0.998217	0.998513	0.998259	BFGS 1428	SOS

## Data Availability

Data are contained within the article.
